# Scaling digital health in low- and middle-income countries: lessons from Malaysia’s cross-sector capacity-building approach

**DOI:** 10.7189/jogh.15.03044

**Published:** 2025-11-07

**Authors:** Jayakayatri Jeevajothi Nathan, Adina Abdullah, Jay Evans, Siti Nurkamilla Ramdzan, Monica Fletcher, Norita Hussein, Nik Sherina Hanafi, Ee Ming Khoo, RESPIRE Collaboration

**Affiliations:** 1 Department of Primary Care Medicine, Faculty of Medicine, Universiti Malaya, Kuala Lumpur, Malaysia; 2 NIHR Global Health Research Unit on Respiratory Health (RESPIRE), Usher Institute, University of Edinburgh, Edinburgh, UK; 3 Nuffield Department of Primary Care Health Services, University of Oxford, UK

## Abstract

The COVID-19 pandemic catalysed the development of digital health interventions across the globe, including low- and middle-income countries (LMICs) such as Malaysia. However, moving from pockets of innovation to sustainable implementation at scale remains a major challenge. This viewpoint presents insights from a digital health training programme using a multi-stakeholder engagement series convened by the National Institute for Health and Care Research Global Health Research Unit on Respiratory Health (RESPIRE) during the pandemic. Through co-designed workshops involving policymakers, healthcare providers, small and medium-sized enterprises, and academic researchers, participants examined systemic barriers to scaling digital health innovations in Malaysia, including issues with infrastructure, regulation, and workforce readiness. We used a home-based pulmonary rehabilitation initiative as a case study to explore these dynamics in practice. Broader lessons include the importance of ecosystem-building, capacity development, regulatory clarity, and inclusive design. Our findings offer transferable insights for strengthening digital health systems in LMICs.

The COVID-19 pandemic catalysed the rapid deployment of digital health interventions globally, including in low- and middle-income countries (LMICs), where health systems often lacked the preparedness and infrastructure for sustained scale-up [1–3]. While digital health presents significant promise in these settings, scaling such innovations remains a persistent challenge due to systemic fragmentation, limited regulatory clarity, and institutional unpreparedness. Understanding and addressing these barriers is crucial to ensuring that digital health tools move beyond pilot stages and achieve meaningful population-level impact.

Malaysia rapidly introduced a range of digital health interventions, including contact-tracing applications, vaccination registration systems, health information dashboards, symptom checkers, and teleconsultation platforms. However, these innovations were mainly opportunistic and therefore failed to scale due to gaps in system integration, regulatory frameworks, data governance, and workforce readiness [4].

As government policies and public health priorities accelerated the shift to remote care, sustaining and scaling telemedicine innovations became essential. While generic video conferencing platforms could have been implemented immediately to scale, they proved inadequate for complex, high-touch services such as pulmonary rehabilitation (PR), which require more interactive and tailored approaches [2]. This highlighted the need to address challenges in scaling digital health initiatives using a systems-thinking approach [3].

## CHALLENGES IN SCALING DIGITAL HEALTH IN LMICS

Despite Malaysia's relatively strong national digital infrastructure, several systemic challenges still limit the effective scaling of health innovations. These challenges fall into four key areas:

### Fragmented ecosystems

While local developers and researchers can rapidly build digital solutions, poor interoperability, information silos, and limited coordination between government, academia, and the private sector restrict long-term integration into national health systems [5,6].

### Policy and regulatory ambiguities

The distinction between informational health applications and regulated medical devices remains unclear in Malaysia and across many LMICs. During the pandemic, emergency authorisations were granted without standard regulatory oversight, raising concerns about quality assurance and accountability [7].

### Health system readiness

Health professionals often lacked the necessary digital competencies to support the transition to virtual care, particularly for complex interventions like chronic disease management, which require sustained engagement and behavioural change [8].

### Design, scalability, and acceptability issues

The urgency to implement digital solutions during the pandemic sometimes resulted in tools that lacked user-centred design, adequate pilot testing, and consideration for long-term sustainability and equity [9,10].

These barriers formed the foundation for our capacity-building strategy, guiding the structure and thematic design of the workshops that followed.

## ADDRESSING SCALING BARRIERS THROUGH CAPACITY BUILDING WORKSHOPS

We delivered a four-part workshop programme series conducted between July and November 2020, purposively sampling participants to ensure representation across key sectors, including clinicians, researchers, policymakers, technologists, and funders. We extended invitations through professional networks, institutional partnerships, and targeted outreach, resulting in 38 individuals attending at least one session.

Each workshop followed a co-design format, combining short plenary presentations, breakout discussions, and design-thinking exercises. We collected data using structured facilitator observation notes, participant reflections submitted through post-session forms, and a feedback survey conducted at the end of the series to assess relevance, utility, and areas for improvement.

We undertook a structured synthesis of facilitator notes, participant reflections, and post-session feedback to summarise key discussion points and identify recurring system-level barriers, structuring in around four workshop themes: innovation, design, regulatory environment, and financing.

## THE THREE-STEP APPROACH

### Curriculum development

A multidisciplinary faculty curated content around four themes: innovation; design; policy and regulatory environment; and finance and funding, chosen for their relevance to sustainable digital health scale-up. We aimed to build a shared understanding of the digital health landscape in Malaysia, while equipping participants to navigate and influence critical drivers of implementation.

### Thematic workshops

#### Innovation

This workshop examined challenges in scaling digital health ecosystems and how COVID-19 realities influenced deployment and design. Participants reflected on their own experiences, identified scale-up support needs, and brainstormed innovation tools for further exploration within the RESPIRE project.

#### Design

This workshop focussed on overcoming design and acceptability issues using the PR tool as an example. Participants explored gaps in digital literacy and infrastructure constraints, generating recommendations for iterative design improvements.

#### Policy and regulatory environment

This workshop addressed classification concerns (*e.g.* medical device *vs.* health information app) and explored appropriate pathways for regulatory alignment. Stakeholders discussed the applicability of existing frameworks and proposed clearer regulatory guidance.

#### Finance and funding

In the last workshop, participants responded to gaps in health system readiness and sustainability, focussing on long-term financing strategies and investment readiness. They mapped available opportunities and developed investment narratives to attract support for a digital health scale-up.

#### Thematic engagement

Facilitated discussions enabled participants to link Malaysia’s digital health bottlenecks directly to the four themes. We grounded these workshops in a real-world use case, the proposed home-based PR application, which provided a practical anchor for applied learning across clinical, technical, and policy domains. Participants shared real-world challenges, explored opportunities for regulatory harmonisation, and discussed sustainable funding and patient-centred design strategies. The workshops fostered continuous dialogue and seeded follow-up initiatives now under way to pilot and refine scalable solutions.

## PR AS A USE CASE

PR is a multidisciplinary intervention that combines exercise, education, and behavioural support for individuals with chronic respiratory diseases [11]. In Malaysia, PR delivery has traditionally been centre-based, with barriers such as travel time, low adherence, and COVID-19 restrictions further limiting access.

The PR intervention highlighted multiple challenges to scaling, particularly user engagement, clinical workflow adaptation, and regulatory ambiguity, while providing a lens to explore feasible digital pathways in LMIC settings [12].

Stakeholders identified challenges across three domains:

1. Patient level – digital literacy, language barriers, socioeconomic disparities, and privacy concerns.
2. Provider level – retraining needs, adapting care delivery, and patient monitoring.
3. System level – unclear regulatory and funding pathways.

Several concrete outputs emerged from stakeholder feedback. For example, design input informed refinements to the PR tool’s user interface, including multilingual options and simplified navigation to accommodate users with lower digital literacy. While direct policy influence was limited at this stage, the engagement process strengthened alignment between research and implementation priorities and built relationships with key stakeholders to support future policy uptake. These outcomes highlight the practical value of iterative workshops in enhancing digital health implementation readiness in LMIC contexts.

## LESSONS LEARNED AND FUTURE DIRECTIONS

Post-workshop feedback revealed high satisfaction (90% rated sessions as relevant and professionally applicable). Participants valued cross-sector diversity and suggested that shorter formats could increase accessibility. We observed no significant divergence across stakeholder groups in terms of satisfaction or applicability of content. Respondents did not report major concerns about applying the learning, though some expressed interest in continued engagement or follow-up support to reinforce knowledge application. These results suggest general alignment across sectors and a shared appreciation for the workshop approach.

More importantly, the workshops highlighted three key enabling conditions for scaling digital health in LMICs. Inclusive stakeholder engagement emerged as essential, while co-design processes helped surface localised implementation challenges and fostered collective ownership. However, achieving genuine inclusiveness and equitable participation in LMIC contexts remains a persistent challenge. Power asymmetries, disparities in literacy and digital access, and the marginalisation of certain communities can hinder meaningful engagement. To mitigate these barriers, future programmes should consider integrating community-based participatory approaches, culturally responsive facilitation methods, and targeted capacity-building initiatives tailored to underserved groups. While we did not include patient groups or marginalised communities in our workshops, several participants had prior experience working closely with these populations, contributing valuable insights to discussions on implementation challenges in underserved contexts. Small and medium-sized enterprises were also represented, although post-workshop reflections noted that institutional power dynamics occasionally constrained open critique. These observations highlight the importance of deliberate facilitation strategies to ensure that all stakeholder voices, especially those less institutionally empowered, are equitably represented in co-design processes. We also identified regulatory clarity as a critical enabler. Early and transparent classification and guidance for digital health tools can reduce uncertainty and support responsible innovation. In Malaysia, the regulatory framework for digital health is still evolving. Digital health tools that meet the definition of a ‘medical device’ are regulated under the Medical Device Act 2012 (Act 737) by the Medical Device Authority (MDA). However, many software-based interventions, such as telemonitoring platforms and symptom checkers, fall into regulatory grey zones. While the MDA has issued a general risk classification guidance document, it does not yet provide detailed regulatory pathways for software as a medical device (SaMD). Compared to international frameworks, such as the World Health Organization guidance on digital health interventions and the EU Medical Device Regulation (EU MDR 2017/745), Malaysia’s current system lacks digital-specific risk stratification and SaMD guidance. These gaps underscore the need for a localised regulatory roadmap, one that aligns with global standards but is adapted to local system capacity and priorities. Moreover, sustained investment emerged as another foundational requirement. Isolated pilot projects, while valuable for proof-of-concept, are not sufficient to drive system-level change. Long-term funding commitments, cross-sector collaboration, and integration into broader health system strategies are necessary to support the sustainable scaling of digital health innovations. Without coordinated and ongoing investment, even the most promising interventions risk being discontinued or underutilised.

The PR tool itself was not scaled due to systemic barriers. These included fragmented institutional support that led to unclear leadership and accountability, short-term funding cycles that prevented sustained iteration and expansion, and regulatory uncertainties regarding classification and compliance requirements. These obstacles, common across LMICs, emphasise the importance of embedding policy alignment and institutional readiness into early-stage digital health innovation. Reflecting on these barriers, several lessons emerged about what might have been done differently. Early efforts to secure sustained institutional leadership could have clarified ownership and accountability. A more robust implementation strategy that aligned the digital PR tool with national health priorities might have helped attract longer-term funding. In addition, closer engagement with regulators from the outset could have pre-emptively addressed classification challenges and accelerated compliance planning. These reflections point to the value of embedding strategic foresight into the co-design and deployment of digital health interventions.

The workshops provided several actionable insights for PR implementation. For example, discussions during the design workshop highlighted the need to simplify the PR app interface for users with limited digital literacy, which led to proposed revisions including icon-based navigation and multilingual support. During the policy and regulatory workshop, participants explored classification challenges specific to PR as a digital therapeutic tool, which informed the recommendation to engage regulatory stakeholders early and seek guidance on whether such tools fall under medical device regulations. These insights helped clarify operational strategies and potential policy engagement pathways that could support future scale-up of digital PR in Malaysia and similar LMIC contexts. The learnings from this case offer a strategic foundation for future digital health interventions aiming for sustainability and equity in LMICs.

## CONTEXTUAL REFLECTIONS AND CONCLUSION

Turning these lessons into action requires concrete next steps across all stakeholder groups. Ministries of health should establish national digital health strategies that include dedicated funding streams, governance structures, and regulatory roadmaps tailored to software-based tools. Academic institutions should integrate digital health implementation research into training programmes and foster collaborative hubs that bridge technology and health sciences. Funders and donor agencies should prioritise longer-term, multisectoral initiatives that support co-design, piloting, and scale-up phases, with a focus on context-adaptive evaluation frameworks. These steps would not only address current fragmentation but also enable sustainable progress in digital health system strengthening.

Digital health holds great potential for LMICs, but addressing its scaling challenges requires a systems-level approach rooted in stakeholder engagement, regulatory clarity, and sustainable investment. The Malaysian experience adds context-specific nuance to global digital health frameworks by highlighting how existing governance fragmentation, linguistic diversity, and uneven infrastructure influence implementation. For instance, the need for multilingual design emerged as a particularly acute barrier in the Malaysian context, underscoring the importance of cultural and linguistic localisation, an area underexplored in mainstream digital health literature. Furthermore, the workshops reaffirmed that policy ambiguity can be especially paralysing when digital innovations intersect with loosely defined device classifications, highlighting the need for localised regulatory dialogues in addition to global guidance.

These insights challenge the assumption that scale-up barriers are predominantly technical or financial. Instead, they affirm the importance of sustained multisectoral collaboration, grounded in local realities. By applying these lessons, LMICs can move from fragmented pilots to robust, scalable, and equitable digital health systems.
